# Two-Spirit Peoples’ experiences accessing and receiving care from community pharmacies

**DOI:** 10.1177/17151635241278751

**Published:** 2024-09-18

**Authors:** Marissa Pirlot, Jaris Swidrovich

**Affiliations:** College of Pharmacy and Nutrition, University of Saskatchewan, Saskatoon, Saskatchewan; Leslie Dan Faculty of Pharmacy, University of Toronto, Toronto, Ontario

## Abstract

**Background:**

Two-Spirit Peoples face unique challenges in accessing and receiving health care in Canada due to health services, including community pharmacy services, being built on hetero- and cis-normative models that impede appropriate care for this group. Currently, there is limited published information on Two-Spirit Peoples’ experiences accessing and receiving care in community pharmacy settings.

**Methods:**

To address the lack of published information, 21 Two-Spirit individuals shared their experiences in a focus group setting. Four different focus groups were held across Canada, including 1 in Saskatoon, Vancouver, Edmonton, and Toronto. Informed by Indigenous methodologies, data were recorded via audio recording and notetaking, and the audio was transcribed and then analyzed for themes using the Voice-Centred Relational Method.

**Results:**

Three major structural systems that affect the experiences of Two-Spirit Peoples in community pharmacies were identified: 1) white supremacy, 2) capitalism, and 3) heteronormativity. These 3 systemic issues presented themselves via racism, homophobia, transphobia, pharmacists’ lack of knowledge about Two-Spirit individuals and their health and lack of time spent educating or building relationships with Two-Spirit Peoples. Participants provided suggestions for how community pharmacists can better serve the Two-Spirit community, such as using inclusive language, adding pronouns and preferred names to patient files, increasing knowledge about Two-Spirit health and advocating for Two-Spirit Peoples.

**Discussion:**

The results suggest that dismantling current structures and ideologies in community pharmacy and society are required to overcome the identified issues.

**Conclusion:**

Two-Spirit Peoples face barriers when it comes to accessing and receiving care in community pharmacies, resulting in many Two-Spirit individuals avoiding health care to save themselves from unsafe and uncomfortable interactions. Pre- and postlicensure pharmacy education about Two-Spirit Peoples is required to improve Two-Spirit Peoples’ experiences accessing and receiving care in community pharmacies.

**Knowledge into Practice**

- Previously, there was no published information on the experiences of Two-Spirit Peoples in community pharmacies.
- Although literature exists regarding Two-Spirit Peoples’ health and health care experiences, this study is the first time Two-Spirit Peoples have been studied in the context of community pharmacy.
- Community pharmacy professionals need to be more accepting of non-Western ways of healing, use nongendered language and educate themselves on what Two-Spirit is.

## Introduction

Colonization in Canada continues to significantly impact the health and well-being of Indigenous Peoples. Racism, heteronormativity, homophobia and transphobia are all embedded in the Canadian health care system, which in turn creates health inequities for Two-Spirit (a term used by some Indigenous Peoples to encompass diverse genders, sexual identities and/or roles) Peoples.^1-6^ An increased risk of suicide and an increased risk of mental health disorders are some of the inequities that this group faces.^2,7^ These embedded harmful systems create inequitable access to health care services for Two-Spirit Peoples.^2,8-10^ Health care services may be inaccessible due to a lack of programming and services for Two-Spirit Peoples, which in turn creates a lack of knowledge and understanding of barriers that impede care for this group.^2,4,8^ They may also be inaccessible due to stresses that this group faces before, during and after receiving care.^11^ To date, there is limited published literature on Two-Spirit Peoples’ experiences accessing and receiving care in community pharmacies. Pharmacists are often considered the most trustworthy and accessible health care professionals by the general public, which should remain true with respect to Two-Spirit Peoples.^12,13^

In addition to the lack of published research on this particular topic, pharmacy schools in Canada generally focus on Western medicines and ways of healing.^14^ This is problematic as it creates a false hierarchy of the validity of various ways of healing.^15^ Of note, there is a lack of Indigenous representation in pharmacy education and practice, which results in current and up-and-coming pharmacy professionals having gaps in their knowledge of the cultural ways of healing for some Indigenous Peoples.^15^ Community pharmacy professionals need to be competent in caring for Two-Spirit Peoples as this group has been made vulnerable by the various systems of oppression in Western society. There is a need for education about, with and by Indigenous Peoples and Two-Spirit Peoples both pre- and postlicensure for community pharmacy professionals, given that the knowledge received in Canadian pharmacy schools is dominated by Western knowledge.^15^ Indigenous knowledge, Peoples, stories and worldviews need to be more visible in the pharmacy profession.

**Mise En Pratique Des Connaissances**

- Jusqu’à présent, aucune information n’avait été publiée sur les expériences des personnes bispirituelles dans les pharmacies communautaires.
- Bien qu’il existe de la documentation sur les expériences de santé et de soins de santé des personnes bispirituelles, cette étude est la première qui porte sur les personnes bispirituelles dans le contexte de la pharmacie communautaire.
- Les professionnels de la pharmacie communautaire doivent mieux accepter les méthodes de soins non occidentales, utiliser un langage non genré et s’informer sur la notion de bispiritualité.

It should also be noted that the various identities of Two-Spirit Peoples play a significant role in the ways that they are able to navigate through society. It is known that the discrimination and inequities faced by a made-marginalized person are greater when there are more made-marginalized identities held simultaneously by that person.^16^ Statistics Canada recently reported the results from a survey conducted between 2014 and 2019, which showed that 33% of Indigenous Peoples experienced discrimination of some form during that time; that number rose to 70% when Indigenous Peoples were also sexual minorities.^17^ This research is important as it looks at Two-Spirit Peoples as their own community rather than grouping them with Indigenous Peoples or the queer community; there are differences experienced in this community that cannot be interpolated through the queer or Indigenous communities alone.^2^

## Purpose/objectives of the research

The objectives of this research project were to listen to Two-Spirit Peoples’ experiences of and ideas about accessing and receiving pharmacy services, to increase Two-Spirit visibility in the health system and across the profession of pharmacy, to ignite national interest among the pharmacy community to engage in knowledge sharing with the Two-Spirit community, and to contribute to improved experiences of Two-Spirit Peoples accessing and receiving care in community pharmacies.

## Methods

Focus groups were used as the research method for this project as they align with Indigenous methodologies and allow for personal narratives to be centred. The focus groups facilitated storytelling sessions for each of the Two-Spirit participants. Recruitment of participants was achieved by collaborating with local Two-Spirit and/or queer organizations in each of the focus group cities, and only Two-Spirit individuals over the age of 18 who accessed community pharmacy services in Canada were included in the study. For the purposes of this study, participants who had a prescription filled at a community pharmacy or interacted with a community pharmacist for the purposes of their health were considered to have accessed community pharmacy services.

The focus groups were facilitated by the authors. The general flow of the focus groups went as follows: a Two-Spirit Elder started the session off in a good way, participants introduced and situated themselves to the group and then participants were asked by the authors: “When it is your turn, please share any stories you have accessing or receiving care in community pharmacies.” After all participants shared their stories, the Elder then ended the focus group in a good way. The focus groups ranged from 82 to 92 minutes in length. All sessions were audio recorded and then transcribed (with all identifying characteristics deidentified) into a transcript and analyzed for themes.

The Voice-Centred Relational (VCR) Method (also referred to as the Listening Guide) was used to code and analyze the data.^18,19^ This method aligns with an Indigenous methodology as a relationship can be formed between the researcher who is coding and analyzing and the participants through their stories, and it allows the researcher to remain accountable in their study.^18,19^ The VCR Method is both a coding and analysis method; thus, the analysis begins during the coding process. The data were coded by the first author, and the analyzed data were shared with the participants for quality assurance. Every participant received the completed transcripts of their individual contributions and was asked if they would like to add, delete or alter any of their stories or anything shared. The main themes that were produced from the participants’ stories were identified in a way that listens to the voices rather than simply hearing them. This project was approved by the Research Ethics Board at the University of Saskatchewan. All participants consented to being in the study and to having the results shared.

## Results

A total of 21 participants gifted their stories to this project. There were 3 participants in the Vancouver focus group, 5 participants in the Toronto focus group, 5 participants in the Edmonton focus group and 8 participants in the Saskatoon focus group. Both positive and negative stories were shared during the focus groups. All stories that were shared had connections to at least 1 of the following themes: racism, time (or lack thereof), corporate greed in community pharmacies, community pharmacy professionals’ knowledge and awareness (or lack thereof) on certain topics and homophobia/transphobia. These results suggest that Two-Spirit Peoples’ experiences in community pharmacies are determined by a multitude of factors such as gender, sexuality, skin colour and institutional processes. In addition to storytelling, some participants offered advice for community pharmacy professionals, which can be found in the implications section. Below are the results from the 4 focus groups.

### Racism

Experiences of racism in community pharmacy settings were plentiful, and the ways in which racism occurred were both overt and covert. Stories were shared about pharmacists assuming individuals were drug-seeking (looking for narcotic or controlled medications), stealing or faking their illnesses. Multiple participants also shared that some community pharmacy health care professionals used racial slurs when interacting with them. It should also be noted that participants shared that they are treated better when they are white-passing. One participant shared how they are treated differently in the winter versus the summer when their skin is a darker colour, and a few participants shared that they see the difference in the way they are treated being white-passing versus their family or friends who look darker than them. These harmful interactions made some patients weary of health care altogether and resulted in some participants avoiding pharmacies, even when they needed to receive care from there.

Additionally, a few participants expressed frustration about having to access multiple pharmacies to pick up all their medications. Two-Spirit participants discussed some community pharmacies not carrying methadone, a medication sometimes used for opioid use disorder. This meant that some participants had to make multiple stops at multiple pharmacies to access all the medications they needed. Participants also described being frustrated with the Non-Insured Health Benefits (NIHB) program. They described being frustrated by not all medications being covered by this program as well as not allowing Métis individuals to be covered by NIHB.

### Homophobia and transphobia

Participants also frequently shared stories about being misgendered and dead-named. They shared how uncomfortable and unsafe they felt when heteronormative assumptions were made about them, when stereotyping took place or when inappropriate language was used. As with experiencing racism in community pharmacies, participants shared that these queer-phobic behaviours made them feel unsafe and uncomfortable in community pharmacies, which also resulted in avoidance behaviours. There were also concerns about access to medications that are used more frequently in the queer community than others, for example, community pharmacies not stocking methadone and some that do not carry HIV medications. Participants from the Vancouver focus group shared that there is 1 main pharmacy in Vancouver where HIV medications are dispensed, so people using those medications must go there. Participants shared that they believe there is a stigma attached to people who use HIV medications, and community pharmacies do not want them in their stores.

### Lack of knowledge

It became apparent that pharmacists’ lack of knowledge in certain areas created poor experiences for Two-Spirit folx. There were stories about pharmacists not being knowledgeable enough when it comes to queer health as well as not being knowledgeable about non-Western ways of healing. Participants shared that they felt respected when pharmacists valued their Traditional Medicines or took time to investigate interactions between their medicines and Western medicines. Two-Spirit participants also expressed frustration with community pharmacists not being knowledgeable enough about applying for drug coverage through NIHB or knowing how to bill NIHB for their medications. A few participants shared that they feel that their community pharmacist is a source of comfort when it comes to the cascade of interacting with health care professionals. One participant shared,

“For the most part, I find (pharmacists) are almost like the last defence for your experience, from seeing a nurse to seeing a physician. And then it’s like, I’ve had extremely bad treatment from them, and I’ve had the pharmacist kind of save that.”

### Time and greed

The final recurring theme of the stories shared centred on the capitalistic nature of community pharmacy. Some participants found that community pharmacists did not take enough time to educate them on their medications, and they left the pharmacy feeling like their health did not matter. They also expressed how frustrating it is when community pharmacy team members shame them by telling them how expensive a medication is. Positive stories were shared when participants found a community pharmacist that they bonded with and kept as their pharmacist for multiple years. In part due to the length of time spent with the same pharmacist but also the care and attention given by the pharmacist to the patient at each interaction, a relationship was formed.

### Implications

Many suggestions to improve experiences in pharmacies were shared. Some include using inclusive language, adding pronouns and correct names to patient files, integrating queer and Indigenous education into pharmacy schools and educating oneself on what Two-Spirit is. The promotion of existing services and programs in community pharmacies also needs to be improved so that Two-Spirit Peoples are aware of the ways that community pharmacies can help provide accessible and timely care. Additionally, participants called for pharmacists to use their position of power to advocate for Two-Spirit Peoples when it comes to NIHB matters and beyond. Below are a few suggestions that participants made regarding improving care in community pharmacies:

“I think it would be good if pharmacists just knew what Two-Spirit was. Just incorporating that into pharmacists’ education.” “We need a place where our medicines are trusted. Where our bodies are respected. Our bodies don’t lie. I think Western medicine and Traditional Medicine can work together; I’ve seen that too. I’m not against Western medicine, but I see that we’re not treated with respect. And dismissed. They don’t see you. You’re invisible. And they just have these conclusions.”

## Discussion

The results of this study suggest there are 3 main structural systems at play that (re)create racism, (re)create homophobia/transphobia, contribute to a lack of knowledge and lead to a perceived lack of time/greed. These 3 oppressive systems include white supremacy, heteronormativity and capitalism, with each being especially harmful and detrimental to groups that experience these systems in multiple ways. The multiple made-marginalized identities that Two-Spirit folx hold position them to be impacted by these 3 systems in more than 1 way, and thus they feel the impacts in a compounded way.

Many suggestions to improve experiences in pharmacies were shared. Three key areas that need improvement include knowledge, accessibility and relationships. Implications that participants shared all relate to these 3 categories. These will be discussed in their respective categories.

### Knowledge

Many participants called for an increase in Indigenous and queer education in pharmacy school curricula. This call also includes education on what Two-Spirit is. Knowledge level in these areas was a major contributing factor to the experiences that Two-Spirit Peoples had in community pharmacies. The lack of knowledge that community pharmacists have about Traditional Medicines is no surprise as Western pharmacy schools do not give as much value to non-Western ways of healing as they do Western ways.^15^ Participants also called for the intentional use of inclusive language as well as adding pronouns and correct names to patient files. It is essential that non-white, non-cis and non-hetero education are included in education to challenge the stigma and discrimination that many Canadian health care professionals hold.^20^ There is an idea that Western medicine is superior to all other medicines in Canadian health care contexts, which is reflected in pharmacy education.^6,14,15^

Not only is there the need to increase the awareness and knowledge of non-Western ways of healing in pharmacy curricula, but there needs to be an increase in Two-Spirit, Lesbian, Gay, Bisexual, Trans, Queer, Intersex, Asexual and other sexual and/or gender minorities (2SLGBTQIA+) education as well. Not only would improving knowledge around these groups improve the health and wellness of them in a physical sense (pharmacists would be able to answer questions related to queer health), but their mental and spiritual health would be improved as well.^21^ Gender and sexual minorities face minority stress, and when health care providers are open, accepting and knowledgeable, this becomes a less stressful environment for them.^11^

Some participants shared positive experiences with pharmacists who did not look down on them for using Traditional Medicines or when they were accepting of queer identities. Although there must be an increase in Indigenous and queer health education in pharmacy schools, community pharmacy professionals can start making meaningful and impactful changes right now by being open-minded and creating a safe and accepting environment for everyone.

### Accessibility

A few issues with accessibility were brought forth by participants in the focus groups. The first issue is the way in which some community pharmacy professionals treated some Two-Spirit participants. Participants shared stories about feeling like a burden or feeling pressured into taking their medications by pharmacy team members explicitly stating how expensive a medication is; this shames and intimidates patients, which makes it less likely for them to want to return to the pharmacy.^22-24^ The promotion of existing services and programs in community pharmacies also needs to be improved so that Two-Spirit Peoples are aware of the ways that community pharmacies can help provide accessible and timely care. The issue of having to make multiple stops at multiple pharmacies to receive all the necessary medications also needs to be rectified.

A final point on accessibility that was brought up during the focus groups is the issue with other health care professionals (physicians, nurses, dentists, etc.) that may be sought before going to a pharmacy. Many participants expressed they have had frequent poor experiences with these health care professionals, resulting in avoiding community pharmacies for fear of further uncomfortable and/or harmful situations. Avoidance of health care settings, including community pharmacies, can create worse health outcomes for patients.^22-24^

### Relationships

The significance of creating and nurturing a positive and healthy relationship between a health care provider and patient is arguably the most important piece to improving experiences for Two-Spirit Peoples in community pharmacies. Not only is the amount of time spent with a community pharmacist important, but the quality of the relationship is just as, if not more, important. Relationships are foundational to Indigenous worldviews.^25^ So, it is necessary not only to create a reciprocal relationship to be accountable and respectful to that person but also to respect that individual’s worldview.

The amount of time spent interacting with a patient is largely dependent on how busy a community pharmacy is. With the ever-increasing capitalistic business models of community pharmacies and multibillion dollar chains operating a significant number of community pharmacies, it becomes more and more difficult to create meaningful relationships. This capitalistic model is not community oriented; it is focused on making money.^26^

In addition to investing time into the relationship, pharmacy team members must ensure that the relationship is respectful in nature. Respect was felt by participants when they were greeted by name, their Traditional Medicines were valued and/or their queerness was openly accepted. Participants shared that they feel safe and comfortable when pharmacists treat them with love, care and attention. When patients feel comfortable and safe in a health care environment, they are more likely to access services when needed.^24^ This alone can create an environment in which Two-Spirit Peoples’ health and wellness are improved.

There are several limitations to this study. Firstly, all focus groups occurred in urban settings, which means that rural and remote voices were not captured. Secondly, only participants 18 years of age and older were able to participate, so youth voices were not heard. Thirdly, the first author is a white person, which may have made participants less likely to openly share their stories. Lastly, the study only heard from 4 locations in Canada. Future studies in this area may benefit from exploring youth voices, rural and remote voices and other urban centres in Canada.

## Conclusion

Two-Spirit Peoples face barriers when it comes to accessing and receiving care in community pharmacies in Canada, which is consistent with other findings in the literature on the health and wellness of this group of people in other health care sectors.^1,2,27,28^ Twenty-one participants shared stories that suggest Two-Spirit folx in Canada face unique barriers when it comes to accessing health care because of the various structural systems that the health care system in Canada is built upon. Heteronormativity, white supremacy and capitalism were identified as the systems of oppression that create poor experiences for this group of people. Participants shared stories about racism, homophobia and transphobia. They also shared that community pharmacy professionals are not knowledgeable enough about Indigeneity or queerness and do not spend enough time with them to build a relationship. These issues result in Two-Spirit Peoples avoiding health care, including community pharmacies. Participants felt positive experiences in community pharmacies when they felt they had a relationship with the pharmacist. The quantity of time with the same pharmacist, as well as a respectful and trustworthy relationship, was valued by participants. Two-Spirit participants called for increased knowledge and awareness among pharmacists and other community pharmacy professionals in the areas of Indigenous health, queer health and Two-Spirit health. ■
